# 3D facial analysis for rare disease diagnosis and treatment monitoring: Proof-Of-Concept plan for hereditary angioedema

**DOI:** 10.1371/journal.pdig.0000090

**Published:** 2023-03-22

**Authors:** Saumya Jamuar, Richard Palmer, Hugh Dawkins, Dae-Wook Lee, Petra Helmholz, Gareth Baynam

**Affiliations:** 1 Genetics Service, KK Women’s and Children’s Hospital, Singapore; 2 SingHealth Duke-NUS Institute of Precision Medicine, Singapore; 3 SingHealth Duke-NUS Genomic Medicine Centre, Singapore; 4 School of Earth and Planetary Sciences, Curtin University, Perth, Australia; 5 School of Medicine, The University of Notre Dame Australia, Sydney; 6 Division of Genetics, School of Biomedical Sciences, University of Western Australia; 7 APAC Rare Disease Medical Affairs, Takeda Pharmaceuticals (Asia Pacific) Pte Ltd, Singapore (at the time of manuscript development); 8 Rare Care Centre, Perth Children’s Hospital, Perth, Australia; 9 Western Australian Register of Developmental Anomalies and Genetic Services of WA, King Edward Memorial Hospital, Perth Australia; University of Waterloo, CANADA

## Abstract

Rare diseases pose a diagnostic conundrum to even the most experienced clinicians around the world. The technology could play an assistive role in hastening the diagnosis process. Data-driven methodologies can identify distinctive disease features and create a definitive diagnostic spectrum. The healthcare professionals in developed and developing nations would benefit immensely from these approaches resulting in quicker diagnosis and enabling early care for the patients. Hereditary Angioedema is one such rare disease that requires a lengthy diagnostic cascade ensuing massive patient inconvenience and cost burden on the healthcare system. It is hypothesized that facial analysis with advanced imaging and algorithmic association can create an ideal diagnostic peer to the clinician while assimilating signs and symptoms in the hospital. 3D photogrammetry has been applied to diagnose rare diseases in various cohorts. The facial features are captured at a granular level in utmost finer detail. A validated and proven algorithm-powered software provides recommendations in real-time. Thus, paving the way for quick and early diagnosis to well-trained or less trained clinicians in different settings around the globe. The generated evidence indicates the strong applicability of 3 D photogrammetry in association with proprietary Cliniface software to Hereditary Angioedema for aiding in the diagnostic process. The approach, mechanism, and beneficial impact have been sketched out appropriately herein. This blueprint for hereditary angioedema may have far-reaching consequences beyond disease diagnosis to benefit all the stakeholders in the healthcare arena including research and new drug development.

## Author summary

The use of 3D facial imaging for clinical assessment has undergone rapid growth in recent years as the associated technical challenges previously hindering its adoption have been overcome. Accurately and objectively mapping the topography of the facial surface allows precise evaluation of an individual’s appearance. This can be very valuable when attempting to characterize the effects of certain diseases or therapeutic interventions. Many rare diseases have been associated with their distinctive facial traits; however, some disorders have more transient impacts on face shape, and as such only qualitative assessment has been available to date. Hereditary Angioedema (HAE) is one such disease; a rare and debilitating genetic disorder that causes frequent severe bouts of swelling mainly in the torso, throat, and face. It is necessary to understand the transient nature of this swelling–particularly in the facial region–to assist with diagnosis and to develop therapies designed to reduce the severity of swelling more effectively. In this report, we detail the specific nature of facial swelling in HAE patients with a view to later analyzing and characterizing the identified traits as they appear in HAE sufferers using 3D facial analysis.

## 1. Rare disease challenges

Rare diseases (RD) resulting from specific genetic variants affect about 6–8% of the population [1]. However, there are >7000 rare diseases, and each of these possess unique characteristics and complexities while posing universal challenges with diagnosis, recognition of disease features, and timely interventions. Delayed or misdiagnosis adversely affects the patients, their care providers, and the healthcare system. Gonzaludo et al estimated that patients suspected of suffering from genetic disorders (GD) in the United States utilized health care facilities more, underwent up to four additional procedures, and their in-hospital stay was longer by 2–18 days, resulting in increased total costs ranging from $12,000 to $77,000 per discharge when compared with patients without a GD associated diagnosis [2]. For example, Noonan syndrome is the second most common GD associated with cardiac abnormalities and the prognosis of cardiac conditions is dependent on the clinical findings on presentation. A retrospective review revealed that the average age of diagnosis is 4.8 years while some get diagnosed in their adulthood [3]. These problems with delayed diagnosis get compounded further for children and families in low-income and middle-income countries (LMICs) due to the scarcity of clinical genetic resources.

## 2. Facial analysis and imaging

Dysmorphic facial features occur in 30–40% of rare diseases, while subtle facial features are present in many more disorders [4]. These unique characteristics contribute to disease diagnosis in varying proportions. A third of all rare diseases, more than 100 million people globally, have subtle facial clues that are recognized as part of the disease phenotype [5]. In recent years different technology-driven elements have been included to facilitate diagnosis. These include digital databases [6], online services [7], analytics software [8], molecular diagnostics, and sequencing. These methodologies remain mainly confined to developed nations. However, despite applying sequencing, almost 50% of patients remain undetected [9]. Non-invasive methods that could enhance clinical decision-making and hasten the establishment of diagnosis are big unmet needs in the quest to better care for patients suffering from RDs. The facial shape corresponding to a syndrome can be disparate, and measuring facial shapes alone or combined with other data could create necessary diagnostic pointers for various RDs and monitoring for their treatment [10,11]. Utilizing standard two-dimensional (2D) facial images for syndrome diagnosis has been applied earlier [4,12,13]. However, three-dimensional (3D) facial images pack in compendious information and more insights than analogous 2D images.

### 2.1. 3D Facial analysis and rare diseases

3D photogrammetry enables the measurement of the spatial coordinates of the surface topography. In a clinical setting, this enables measurement and analysis of the whole undistorted face of the person, and each of the components and areas comprising the face and any other parts of the body that have clinical significance in diagnosing, or by recording the face, and facial features over time, to be able to accurately measure and monitor change in the condition objectively over time. This can be applied to objectively assessing treated and untreated natural history in a personalized manner.

Deep phenotyping of facial dysmorphology with 3D photogrammetry in genetic syndromes has been deployed for patients suffering from Loeys-Dietz syndrome and Shprintzen-Goldberg syndrome [14], Ectodermal dysplasia [15], plastic surgery [16], and Congenital Adrenal Hyperplasia [17] etc. Hallgrímsson et al studied variations in facial images of 7057 subjects (3327 with 396 types of syndromes, 727 of their relatives, and 3003 unaffected, unrelated subjects). Machine learning and parametric approaches were developed to arrive at automated syndrome diagnosis using 3D facial images. Additionally, the work shed light on the possibility of identifying unrecognized cases or semi-dominant inheritance by applying 3D facial imaging on “unaffected” relatives [18]. Outside of the domain of RDs, Ali et al collected 1812 videos of 604 individuals—61 with Parkinson’s disease (PD) and 543 without PD through a web-based tool and analyzed the facial action units (AU). The facial micro-expressions prediction accuracy was comparable to motor symptoms utilization methodologies. The Support Vector Machine trained on the variances for the automated classifier achieved 95.6% accuracy thus paving the way for the utilization of facial expressions as a future digital biomarker for PD [19].

3D photogrammetry shows the promise to become an expert system for RD syndrome diagnosis. Since the technology is in a nascent stage for clinical research creating evidence through quantitative frameworks and applying these principles to classify facial shapes into syndrome categories with mild, moderate, and high accuracy is necessary. In this review, we endeavor to present the proof of concept rationale for effective diagnosis and disease progression in Hereditary Angioedema (HAE) by applying 3D photogrammetry and Cliniface software. We also intend to explore the opportunities to validate these findings first in high-income countries and subsequently transfer the clinical know-how to scale up and apply in a culturally safe manner to their respective populations, in LMICs.

### 2.2. 3D facial imaging vs 2D photography

3D photogrammetry supports a wider variety of measurement modes than 2D photography. This includes measurements between points directly and over the curved facial surface as well as angle and curvature measurements. The key advantages of 3D imaging, when compared with 2D images, are the following [20]:

- Increased precision
- Ability to capture and provide absolute and relative measurements
- Eliminates problems of the pose, or angle of the subject
- Abrogates the significant lighting issues, and related artefacts, inherent to 2D imaging

3D imaging provides significant real-world utility for clinical studies compared with 2D imaging which produces visual distortions. The image data captured is reproducible and provides data with exceptionally fine analytical accuracy. These qualities ensure a high degree of quantitative confidence and statistical validity, in any changes detected in an individual over time, or variation from comparison with normative population data. The benefits are particularly useful in clinical settings where the pose of the patient or the inflection of their head is fixed by skeletal or neuromuscular symptoms of particular diseases; or for new-born babies and children where pose, muscle control, and strength imply the head and angle of the face can vary greatly; and in the real-world clinical settings where images are captured in different locations and varied lighting conditions. These issues are all mitigated with 3D image capture, as the precision of the data enables each image to be rotated into a standard pose, with systematized reference points of the face enabling comparative analysis; and shadows from lighting removed without affecting the integral data accuracy in the images.

### 2.3. Cliniface software—Analysis of 3D images

Cliniface [20] is a desktop software application that provides a suite of tools to visualize, measure, and analyze 3D facial images. Cliniface can automatically place landmarks on a face, as well as extract and show standard facial measurements. At any stage, a clinician can interact with the software and for instance, improve the location of the extracted facial landmark. The landmarks are then used to make inferences about atypical features of clinical significance, that when used together with other phenotypic information about a patient may assist in rare disease diagnosis. Other fields of application are clinical trials, and treatment monitoring, surgical planning and audit, and design and audit of personalized facial devices [21]. Cliniface has a unique combination and a growing network of support from across the research, medical, community, and commercial sectors, with more than 4000 software downloads and implementations covering 4 continents [22]. Herein, we consider the potential applications of 3D facial analysis in the realms of a treatable rare disorder—HAE.

## 3. HAE: Presentation, diagnostic odyssey, and therapeutic challenges

HAE is a complex, rare, and autosomal dominant disorder caused by mutations in *SERPING1* resulting in a lack of or a dysfunctional C1-inhibitor protein [23]. Symptoms of HAE typically begin in childhood, may worsen during puberty and perimenopausal years, and continue throughout an individual’s life. It presents with recurrent attacks of severe swelling, and angioedema, that may involve any part of the body and occur in unpredictable frequency, the severity of pain, degree of swelling, and site. Symptoms of HAE range from mild to severe and, when severe, can result in hospitalizations or lead to life-threatening complications. Attacks may involve the face, lips, tongue, throat and larynx, gastrointestinal tract, and the genitourinary system, as well as the limbs [24]. Symptoms typically begin in childhood and continue lifelong, resulting in a significant burden of disease on patients, their families, and the health system [25].

Treatment for HAE includes managing the attack acutely and preventing recurrence with the use of C1 esterase inhibitor prophylactic therapy. However, despite the advances in diagnostics and therapeutics, the ongoing burden of disease and the significant unmet needs of people living with HAE were highlighted in the USA FDA survey [23] in which two-thirds of patients, despite their use of prophylactic medications (primarily C1 esterase inhibitor products or androgens) over the previous year(s), still experienced a high frequency of attacks, and a high rate of anxiety and depression. They also reported symptoms that often led to a high rate of health care resource use, interfered with patients’ daily lives, and caused work and activity impairments. Symptoms are typically at maximum intensity for up to 24 hours before spontaneously resolving in a further day. [26,27].

In addition, there are persisting diagnostic delays [28,29] contributing to a high burden of disease that markedly impacts Quality-of-Life of the person and their family [30,31]. Delays in diagnosis are also a recognized high cost to the health system in terms of unnecessary outpatient and hospitalizations and impact on resource allocations [29,32]. Several diagnostic and prognostic biomarkers have been identified. However, their analytical validity, clinical utility, and applicability in various stages of the patient journey remain unfulfilled [33]. The severity, manifestation, and progression have been found to differ even amongst the family members with similar mutations [34].

### 3.1. Stakeholder perception mapping on HAE—Identifying needs and gaps

This paper utilized mixed methodological approaches including a desk-top literature review of clinical academic publications, information from patient organizations, and published stories from patients living with HAE. These information sources were further augmented through semi-structured interviews with internationally recognized clinical experts for HAE and interviews with people who had lived experiences of the condition. This approach provided insights into the real-world burden of disease currently being experienced by the patients and their families, and identified the current real-world priorities of clinicians and patients in better treating and managing their disease. People living with HAE were also asked to describe in their own words their awareness, or otherwise, of any facial symptoms and traits that they believed were associated with their condition.

**Fig 1:**
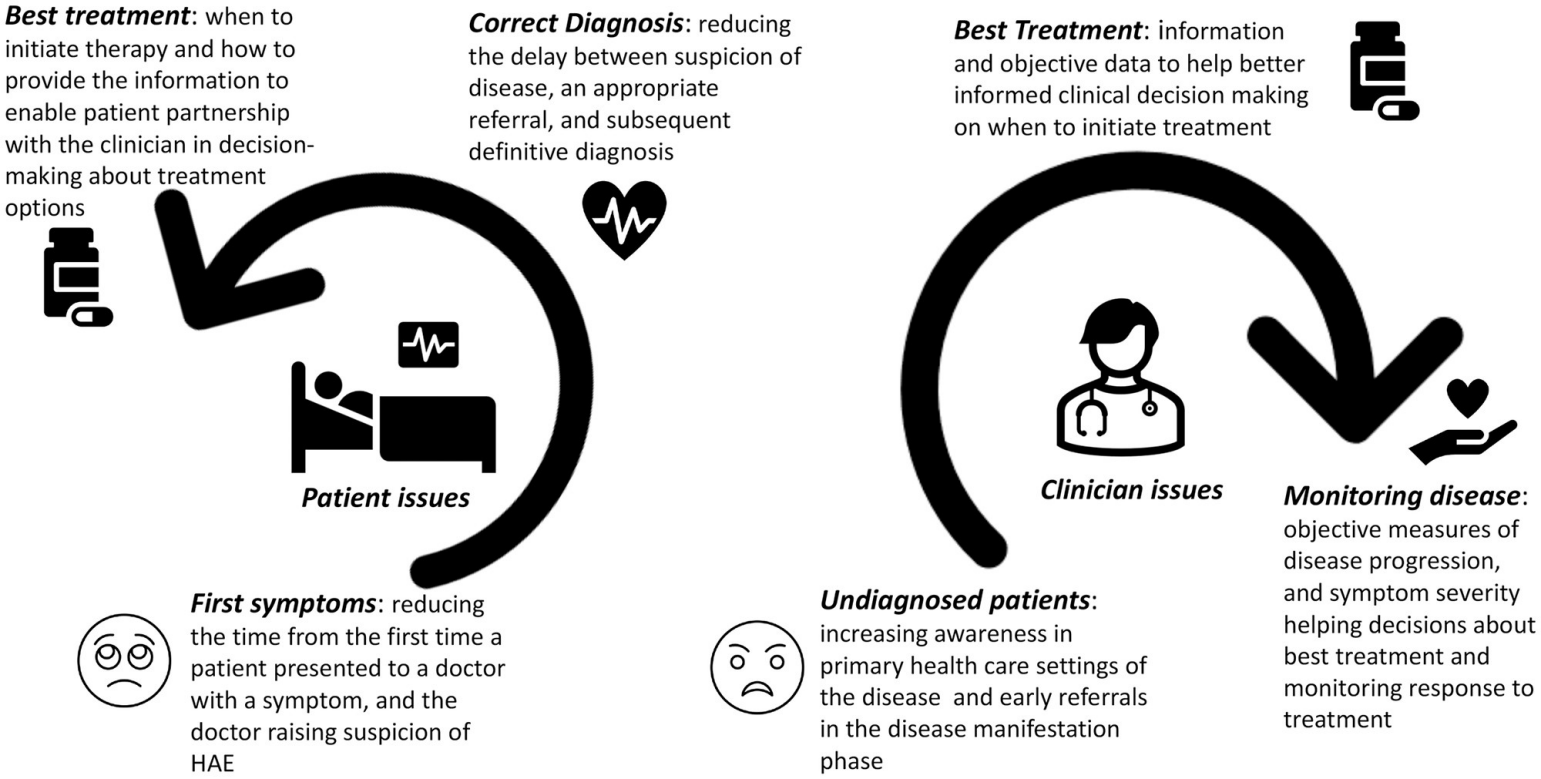
Key issues reflected different perspectives and consequential subjective differences in weightage to priorities applied by clinicians and patients. A universal roadmap emerged through the consultation process, specifically that the QoL issues were integral to optimizing outcomes and reducing the overall impact of the burden of disease for both the stakeholders—the patient and the health system.

The obtained insights were used to build a subjective ranking of issues (Fig 1). The subjective rankings were an aggregate of weightage based on individual perspectives—be it a clinician or a patient living with a rare disease. The priorities were assessed thematically around the impact of diagnostic delays and the emotional, social, and economic burden of disease, including Quality-of-Life (QoL).

### 3.2. HAE 3D facial analysis hypothesis

HAE pathogenesis mechanism involves fluid accumulation in the interstitial space of various tissues through noninflammatory and nonallergic processes. Clinical features include sudden onset of swelling on the face (lips, around eyes, tongue) and extremities. Diffused skin edema has ill-defined margins and is non-pitting in nature. The subtle changes in facial contours due to fluid accumulation as a part of disease manifestation could provide vital clues to the onset of an acute trigger. Also, the subsiding of swelling on the face (and other affected areas) in response to the treatment may offer key insights on individual responsiveness as well as predicting the prognosis in relative measures. The authors believe that solution may exist in capturing and identifying the onset of edema signs in the very early initiation of disease pathogenesis. By blending human intelligence and technology benchmark the early clues of HAE and differentiate them from closing mimicking conditions of defined or known etiology.

There is a need for objective tools to assist early diagnosis and that support decision-making on treatment initiation and regime. Integral to these needs is the ability to be able to better monitor disease progression and treatment outcomes. These findings have been used to propose a disorder-specific hypothesis that needs to be investigated, and with this improved knowledge base, provide an opportunity to be translated into clinical tools to improve diagnosis, and identify better objective biomarkers of treated natural history and treatment response.

**Fig 2:**
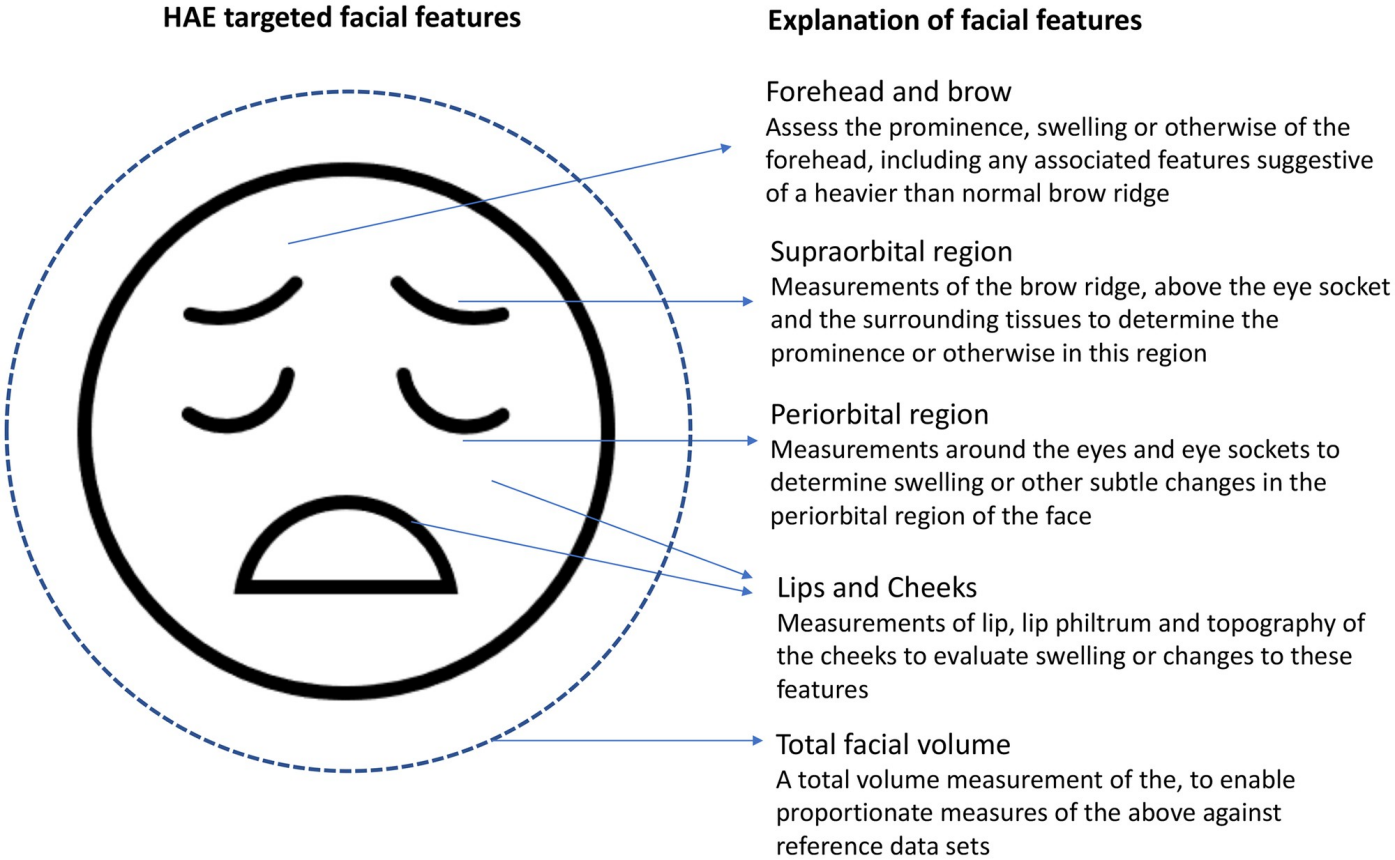
Features and importance of various facial elements and their resultant impact from people living with HAE amenable to Cliniface 3D photogrammetric analysis. Subtle or drastic changes owing to edema.

The rationale that forms the basis for considering 3D facial analysis, is that people living with HAE have facial signs (facial phenotypes) whose precise and objective assessment (Fig 2) may lead to:

- raising the index of suspicion of the disease leading to earlier diagnosis
- objectively monitor the treated and untreated natural history, including identifying the early evolution and resolution of episodes

**Fig 3:**
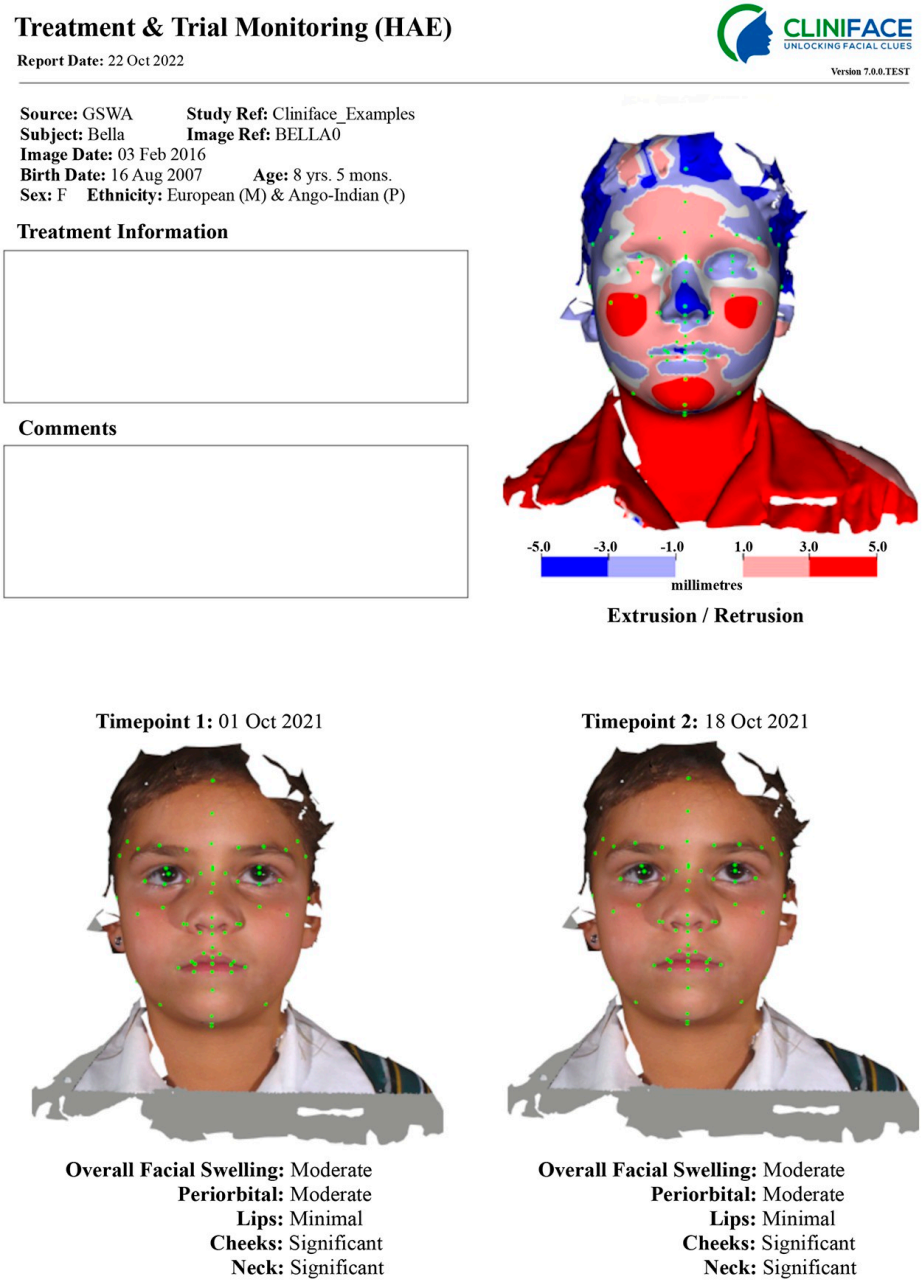
Mock-up example of Cliniface 3D facial ‘Hereditary Angioedema treatment and monitoring report’ Parental consent was obtained from the parent (Gareth Baynam) of the child whose image is appearing in Fig 3 who is also the corresponding author.

The objective of exploring HAE is to investigate the use of tools that can objectively monitor 3D facial signatures (Fig 3) that might improve patient outcomes. Specifically, through

- diagnostic assistance (e.g. early diagnosis of HAE and individual episodes)
- provide specialists and primary care providers, and patients an avenue to better monitor and manage their conditions and their treatment

## 3.3. Outcomes and resultant impact with 3D photogrammetry in HAE

**Fig 4:**
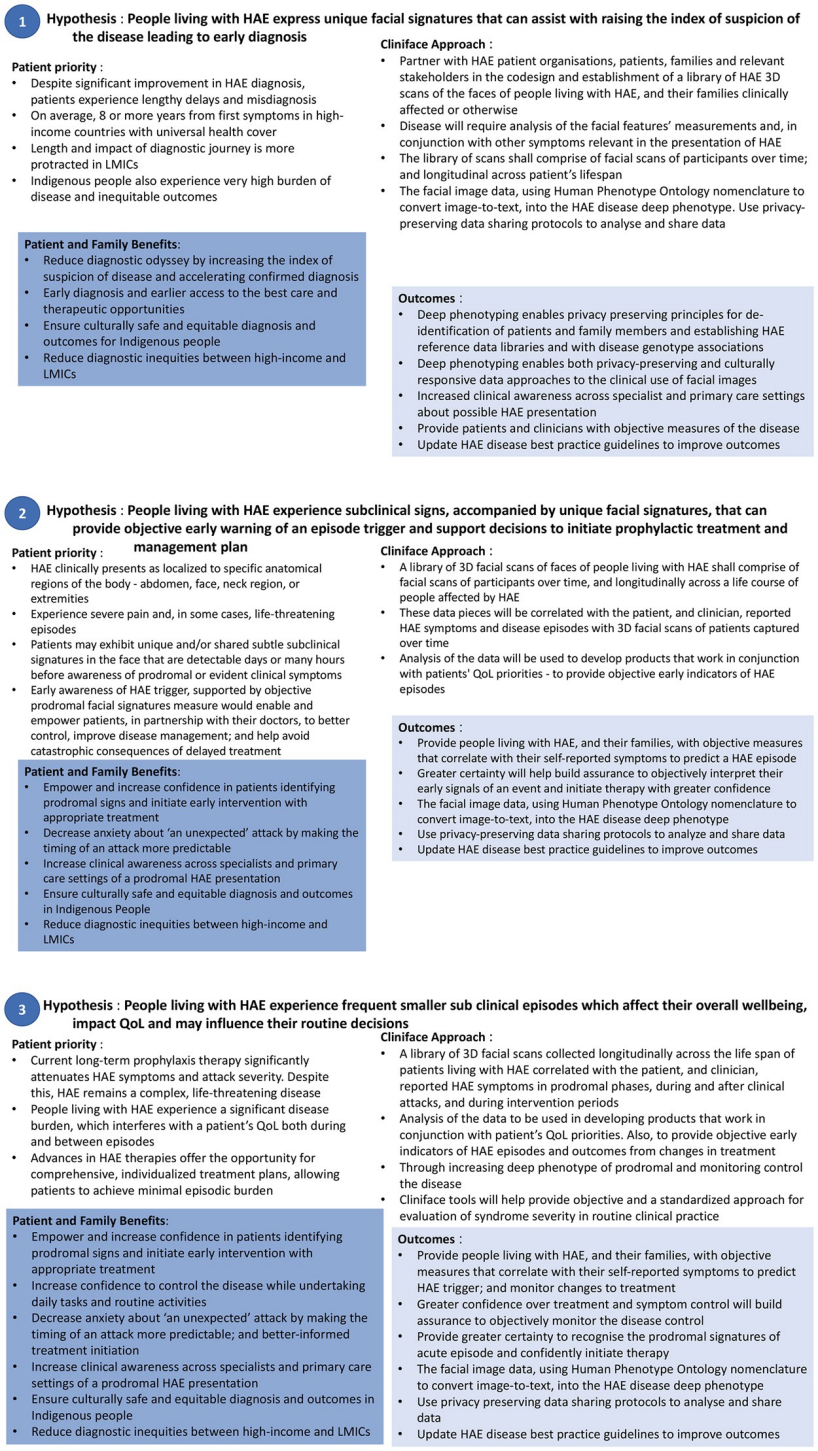
(1) Increasing index of suspicion and improving early detection and diagnosis of HAE. (2) Early objective prodromal indicators of an imminent HAE episode. (3) Improve treatment outcomes and QoL for patients living with HAE.

Early disease detection and syndrome identification by analyzing physiognomy through quantitative 3D facial imaging have great potential. Potential outcomes and the impact of 3D facial analysis in the HAE patient journey have been enumerated herewith. The authors present 3 potential hypothetical scenarios herein that map patient needs and while calibrating these vital needs with Cliniface solution they endeavor to identify possible outcomes resulting in overall societal benefits to all stakeholders with special emphasis on patients and their families. These are presented and grouped by the following:

- index of suspicion and diagnosis (Fig 4(1))
- early prodromal indicators of a disease episode (Fig 4(2))
- improving treatment outcome and quality of life (Fig 4(3))

**Fig 5:**
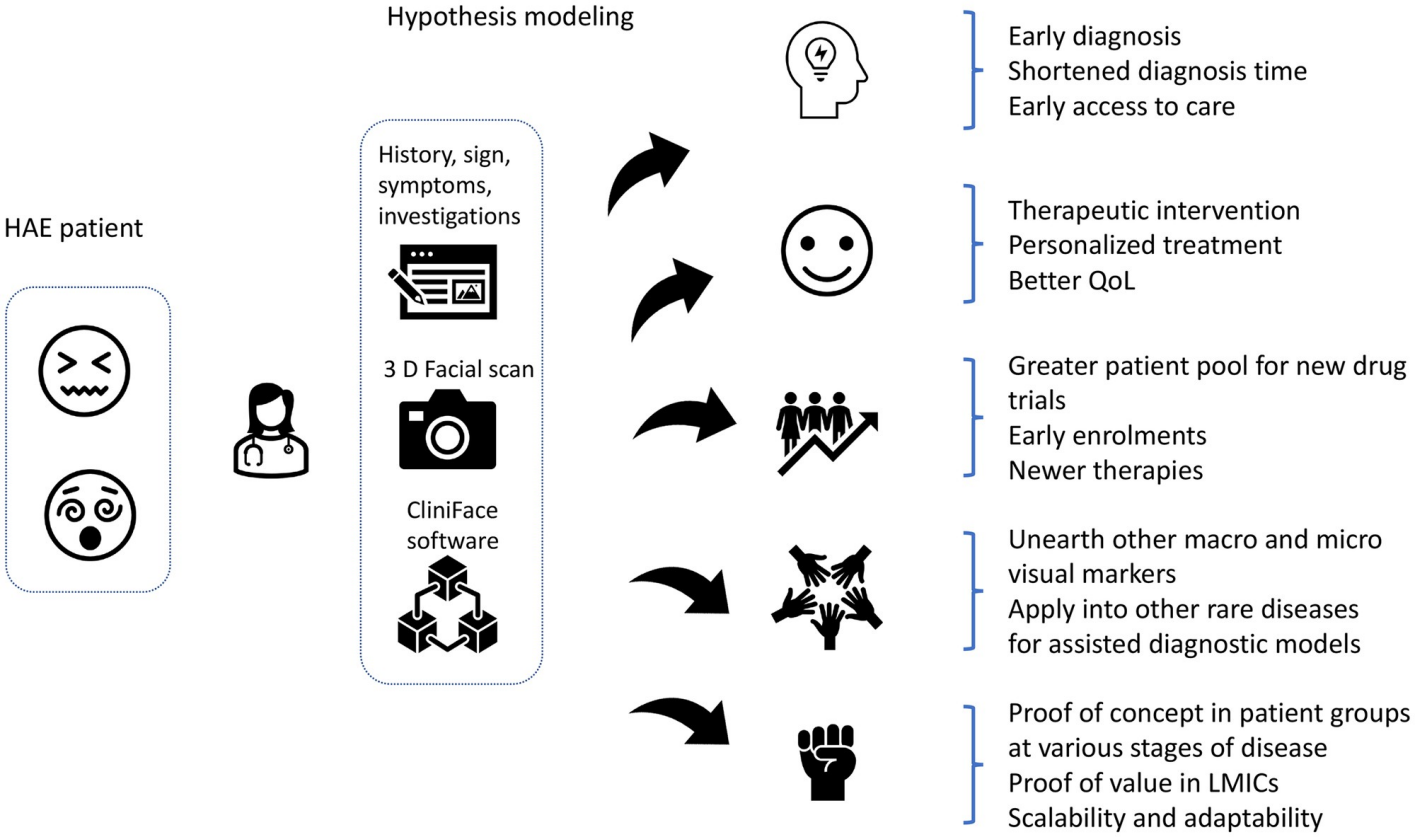
Hypothesis benefit model in HAE patients with 3 D facial scan and Cliniface software.

There is a tremendous opportunity to build a knowledge base of 3D facial data, and correlate these with clinical data and patient-centric metrics for the overall benefit of all the stakeholders in the care delivery spectrum. Patient-reported outcome measures (PROM) and patient-reported experience measures (PREM) could be additional parameters to establish digital biomarkers [35] for HAE. Our hypothesis model (Fig 5) illustrates additional benefits of facilitating new drug development, clinical trial recruitment and the opportunities to extend the model to other rare diseases.

## 4. Novel opportunities from the proposed Cliniface initiatives

Through the review and consultation process, several new opportunities have become evident for 3D facial analysis to provide value to HAE patients and clinicians including:

- 3D facial scans that include positioning and pose to capture the neck of the patient may provide objective detection of unique or shared subtle subclinical signatures in the face that correlate with facial and laryngeal manifestations. Objective measures that might alert a patient to avert a traumatic or potential exacerbation.
- Objective 3D facial assessments can be used to inform the creation and assessment of personalized facial devices to increase the quality of life e.g., partnering with 3D printing for improved management of sleep apnoea. It is noted that this may be less relevant to HAE, and more relevant to many other rare and common disorders.
- Expanding the scope of Cliniface to include 3D scans of a patient’s abdomen would provide objective reproducible measurements of swelling to support pain scores and other symptoms to better define the deep phenotype of HAE and contribute new knowledge to better understand and treat HAE. Current 3D imaging hardware, and measurement tools in Cliniface support the technical feasibility of implementing this approach.

## Conclusion

This exploratory proof of concept hypothesis sets out to identify facial traits and respective objective measurements that are likely to be associated with HAE as a foundation to improve diagnosis, and with this improved knowledge base, progress towards better objective markers of treatment response. Through this mixed-methods investigation, several facial traits can be identified for exploration and testing in future studies to improve the quality of life for patients and reduce the burden on the health system.

The Cliniface software is a globally applicable and accessible solution for supporting diagnosis; treatment monitoring; and novel assessment of disease complications, such as sleep apnoea [36]. The existing Cliniface approaches are tailored to different population groups (e.g. Aboriginal Australian reference data, Czech reference data, mixed ethnicity reference data, newborn to adulthood reference data). There are ongoing additions of e.g. Singaporean (comprising of different distinct and mixed ethnicities), Chinese, and Japanese reference data sets to be able to create more dispersed and diverse applications. The 3D technology is non-invasive, portable, and supports patient engagement with co-design approaches. Additionally, the ability to collaboratively develop software solutions and obtain insights from a diverse population mix within the ambit of jurisdictional regulations (*e*.*g*., identifying facial scans do not need to be shared) while allowing privacy-preserving data sharing (*e*.*g*., through face-to-text conversion) further support international partnerships, equity, inclusion and scaling.
